# Influenza Virus: Global Health Impact, Strategies, Challenges, Role of Nanotechnolgy in Influenza Vaccine Development

**DOI:** 10.3390/vaccines13090890

**Published:** 2025-08-22

**Authors:** Shabi Parvez, Anushree Pathrathota, Arjun L. Uppar, Ganesh Yadagiri, Shyam Lal Mudavath

**Affiliations:** 1 School of Pharmaceutical Sciences, IFTM University, Moradabad 244102, Uttar Pradesh, India; 2 Department of Pharmaceutics, JSS College of Pharmacy, JSS Academy of Higher Education & Research, Mysuru 570015, Karnataka, India; 24p08002@jssuni.edu.in; 3 Department of Pharmaceutics, BLDEA’s Shri Sanganabasava Mahaswamiji College of Pharmacy and Research Centre, Vijayapura 586103, Karnataka, India; arjunuppar2001@gmail.com; 4 Department of Pharmacology & Toxicology, National Institute of Pharmaceutical Education and Research (NIPER), Kolkata 700054, West Bengal, India; yadagiriganesh@gmail.com; 5 Department of Animal Biology, School of Life Sciences, University of Hyderabad, Prof. C.R. Rao Road, Gachibowli, Hyderabad 500046, Telangana, India

**Keywords:** influenza, vaccination, nanoparticles, infections, immune response

## Abstract

Influenza is a serious and global health issue, and it is a major cause of morbidity, fatality, and economic loss every year. Seasonal vaccines exist but are not very effective due to strain mismatches, delays in production, and antigenic drift. This comprehensive overview discusses the current situation of influenza vaccination, including the numerous types of vaccines—inactivated, live attenuated, and recombinant vaccines—and their effectiveness, efficacy, and associated challenges. It highlights the effects of the COVID-19 pandemic on the trends of influenza vaccination and the level to which innovation should be practiced. In the future universal influenza vaccines will be developed that target conserved viral antigens to provide long-term protection to people. In the meantime, novel vaccine delivery platforms, such as mRNA technology, virus-like particle (VLP), and nanoparticle-based systems, and less cumbersome and invasive administration routes, as well as immune responses are also under development to increase access and production capacity. Collectively, these innovations have the potential to not only reduce the global influenza epidemic but also to change the way influenza is prevented and prepare the world for a pandemic.

## 1. Introduction

Influenza is an acute viral respiratory infection that can break out and potentially cause a pandemic or epidemic [1]. These infections annually cause 250,000–500,000 fatal cases and 3–5 million clinical infections. Half a million people die annually due to the influenza epidemic [2]. ‘Flu’ is a commonly used term for influenza, which is a highly infectious respiratory infection caused by the influenza virus and has a shorter incubation period of 1–4 days. The clinical manifestations include headache, body chills, fever, body aches, and cough. However, people usually recover from this infection, except in cases like pregnancy, old age, infants, and those with chronic health problems. Influenza is a respiratory illness that can be mild or severe, leading to hospitalization or even death [3].

Influenza viruses are an *Orthomyxoviridae* family negative-sense single-stranded RNA virusand classified as influenza A, B, C, and D [4]. Seasonal influenza epidemics are prominently due to infection with the Influenza B and influenza A viruses of H1N1 and H3N2. The Spanish flu (1918) outbreak was due to the influenza A virus of the H1N1 subtype. The Asian flu, in 1957, caused by the influenza A H2N2 strain, was replaced by the H3N2 subtype. In 1977, the H1N1 influenza A caused a pandemic, and again in the 21st century, a new influenza A virus H1N1 caused a pandemic of swine flu origin [5]. Ninety-nine percent of deaths in children younger than 5 years with influenza-associated lower respiratory tract infection occur in developing countries [6]. The emergence of a new strain of the influenza virus for which humans do not have any immunity causes the occurrence of an influenza pandemic. Severe illnesses occur due to person-to-person spread of the novel virus around the globe [7]. In 1918, the deadly influenza outbreak took approximately 50 million people’s lives, which was more than the whole of World War I (1914–1918). Two major pandemics of the 20th century, 1957 and 1968, killed over 1 million people each. The H1N1 (2009) pandemic claimed 575,000 lives, not only those who were already sick with other illnesses, but also healthy people [8]. In 1940, Influenza B emerged and was identified in respiratory illness in children, which was less common as compared to Influenza A. Influenza B has no animal reservoir and is not associated with a risk of pandemic [9]. With low prevalence, typically with mild symptoms, the influenza C virus does not figure in routine virological investigation [10]. Immunization is the least costly and most dependable way of preventing infections [11] and serious issues concerning the influenza viruses [12]. Regulatory analysis requires evaluation of immunogenicity and evaluation of efficacy of the vaccine in the case of influenza vaccines. Randomized controlled trials monitor the immune response, which is called immunogenicity, and the capacity of a vaccine to stop illness is referred to as vaccine efficacy. Furthermore, the efficiency of vaccines is assessed in real-world settings via observational evaluation, usually by a test-negative design to estimate results like the number of hospitalizations/mortality rates. The ideal strategy for determining efficacy is a double-blind, controlled, and randomized design, as it is essential to analyze collected data evidence using the standard method [13].

Due to the constant evolution of influenza viruses, routine modifications to influenza vaccine formulations and global monitoring are necessary. For five decades, the World Health Organization has worked with scientists and policymakers to establish a framework for international influenza vaccine manufacturing and evaluation, as well as regulatory oversight of vaccine distribution. The constant evolution of influenza viruses requires global surveillance to create novel vaccine formulations. To recommend seasonal influenza vaccine strains for distribution in the northern and southern hemispheres, the WHO holds two annual technical meetings in February and September. Data gathered via the Global Influenza Surveillance and Response System (GISRS) of the World Health Organization (WHO) served as the basis for the recommendations. GISRS has expanded its scope since 2004 to include A (H5N1) and A (H9N2) influenza subtypes, which has improved pandemic response readiness. The process of developing high-yield candidate vaccine viruses combines the expertise of WHO Collaborating Centres and specialized laboratories, which may employ classical reassortment, established in 1971, or reverse genetics methods. Authorized organizations receive the characterized candidate viruses that the WHO creates through its laboratories. The production of reference materials for global distribution occurs through Essential Regulatory Laboratories, which work together with vaccine manufacturers to deliver their product [12].

In March 2024, a significant epidemic of the H5N1 avian influenza was discovered in the dairy cattle herds of the United States, the first outbreak of this phenomenon in the bovine population. The epidemic peaked on March 31 and remained with 121 new cases in a herd of 3876 cows, but most of the infections (89.4 percent seroprevalence) were subclinical [14]. Any zoonotic danger was accentuated by human infection that was first documented in Texas on April 1 [15]. Cows affected had diminished production of milk over a period of more than two months. Due to potential cross-species transmission and reassortment, nanovaccine platforms, virus-like particles, nanoemulsions, and chitosan-based and DNA nanovaccines are in development, as they can induce strong mucosal and systemic immunity and prevent transmission through the utilization of conserved antigens, such as M2e [16].

This review article highlights the insights into the influenza virus’s impact on global health and strategies for vaccine development. It also focuses on the challenges in the development of vaccines against influenza, as well as the role of nanotechnology in vaccine development.

## 2. Influenza Immune Response

**Figure 1:**
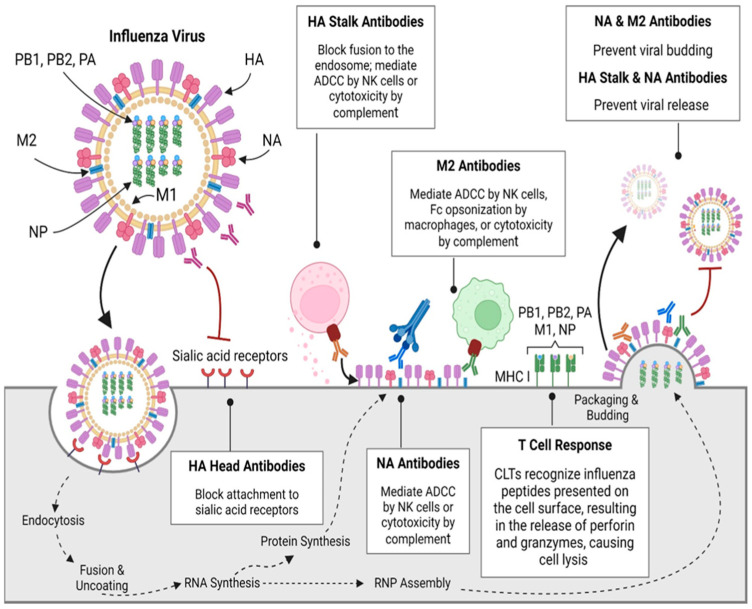
Immune responses to influenza viruses. Abbreviations: CD8, cluster of differentiation 8; PA, polymerase acidic; PB1, polymerase basic protein 1; PB2, polymerase basic 2; RNP, ribonucleoprotein [17]. Adapted with permission from copyright © 2024 The Author(s). Published by Elsevier.

The complex chain reaction of humoral and cellular immune responses is a crucial event in a protective immune response that wards off infection and prevents the emergence of serious illness, as shown in Figure 1. Evaluating influenza vaccines (licensed) and the new vaccine development aiming to prompt such defensive responses requires an understanding of the functions of each component of a protective immune response, the mechanisms by which they are prompted, and how they vary among populations.

### 2.1. Antibody-Mediated (Humoral) Response

Infection caused by the influenza virus induces specific antibody responses against the virus antigen [18]. CD4+ T cells play a vital role in aiding B cells in the production of antibodies and activate, proliferate, and differentiate into T cells to interact with B cells. Upon this interaction, along with cytokine signaling, B cells will be converted to plasma cells. The plasma cells are responsible for making highly specific antibodies for the influenza virus [19]. The first line of defense of the body is provided by antibodies, which attach to viral antigens and stop their action. These antigens contain two surface proteins: (i) neuraminidase (NA), which is present on the virion, as well as the host, and (ii) HA, which is the key target of the majority of influenza vaccines. Antibodies can also spread to the portions of the ribonucleoprotein (RNP) and the influenza A Matrix 2 (M2) ion channel on the surface of infected host cells. However, 80% of the viral surface is made up of HA, a predominant glycoprotein [20,21]. Antibodies against HA have been accepted as a correlate of protection (CoP) [22]. In a vaccine clinical trial, seroconversion is considered an immunological endpoint. Mostly, the HA-neutralizing vaccine targets the globular head domain. Despite the high specificity of antibodies and their ability to block HA and sialic acid binding, hindering cellular entry, the globular head domain displays significant variability due to antigenic drift, leading to insufficient cross-reactivity among strains. But antibodies against the HA head region and stalk domain have shown cross-reactivity, making them ultimate targets for the universal influenza vaccine [23]. HA stalk antibodies can coordinate Fc receptors’ effector functions as antibody-dependent cell cytotoxicity (ADCC), antibody-dependent cellular phagocytosis (ADCP), and activation of the complement pathway [24]. Epitope masking occurred due to a head-specific response and primary targeting of the variable head epitopes by the immune system over the conserved stalk epitopes [25]. NA has reduced immune selection pressure and exhibits only a limited number of subtypes, with a slow antigenic drift rate. Therefore, antibodies generated after natural infection targeting NA display cross-reactivity against several influenza strains of the identical NA subtype, along with the original strains of the pandemic (H1N1 and H3N2). Natural influenza infections have an antibody response against HA and NA [26]; however, NA-based immunity does not inhibit infection but only reduces viral titers in the viral shedding, lungs, and severity of the disease [27].

### 2.2. Cell-Mediated Immunity

Cellular immunity refers to the response mediated by T cells. T cells are produced from the bone marrow and the fetal liver, then are transferred to the thymus for further maturation and the development of their T-cell receptor (TCR). Cell-mediated immunity (CMI), predominantly facilitated by CD8+ T cells (cytotoxic T lymphocytes (CTLs)) [15], plays a vital role in the innate defense of the body against influenza. CTLs are associated with minor viral shedding by eradicating infected cells, resulting in a decreased viral load [28]. CD8+ T cells inhibit viral replication by producing pro-inflammatory cytokines, interferon (IFN)-γ. CD8+ T cells protect against the progression of serious diseases, even in instances of circumvented antibody-mediated immunity [29]. The CD4+ T cell subset, CD4+ CTL, is known for its cytotoxic activity, which provides perforin-dependent protection [30]. Th1 and Th2 (CD4+ T helper cells) have important and complementary roles. For defense against intracellular infections, Th1 cells prominently produce crucial cytokines, such as interferon-gamma (IFN-γ) and tumor necrosis factor alpha (TNF-α) [31]. Macrophages and cytotoxic T cells are activated on Th1 responses for viral clearance and produce IgG isotypes (IgG1 and IgG3) for opsonization, while IL-4 and IL-5 (Th2 responses) support B cell activation and produce IgG4 and IgE, aiding in defense against extracellular pathogens and toxins. The balance between Th1 and Th2 responses is key to effective immunity against influenza [32].

Pattern recognition receptors (PRRs), including toll-like receptors (TLRs 3, 7, and 8), play a crucial role in the immune response to influenza by recognizing viral RNA. Upon detection, they activate dendritic cells and macrophages, leading to the production of pro-inflammatory cytokines and type I interferon. This enhances the effectiveness of CD8+ cytotoxic T cells and CD4+ helper T cells, aiding in the clearance of infected cells and strengthening the overall immune response against the virus [19]. The internal viral proteins (nucleoprotein and matrix (M1) protein) present on the Major Histocompatibility Complex (MHC-I) after infection, trigger a response from CD8+ T cells, and have a protective role in the immunization strategy [33].

### 2.3. Mucosal Immunity

Mucosal immunity is a protective immune response involving the respiratory mucosa passageway for influenza, which involves antibodies and cellular constituents to halt invading viruses. Before entering the systemic circulation, at the mucosal barrier, secretory immunoglobulins (S-IgA) eliminate the virus without activating the complement pathway. In this way, cytokine storm could be controlled, and severe complications due to the activation of pro-inflammatory responses can be avoided [34]. Tissue-resident memory cells (TRMs) of the respiratory tract, like CD8+ TRMs, are also an integral part of mucosal immunity against influenza [35]. Inhaled influenza virus can be eliminated by CD8+ TRM cells present in the upper airways and in bronchus-associated lymphoid tissues (BALT), which protect the body after reactivation by rapidly producing key cytokines (IFN-γ and TNF-α).

## 3. Vaccination Strategies for Influenza

**Figure 2:**
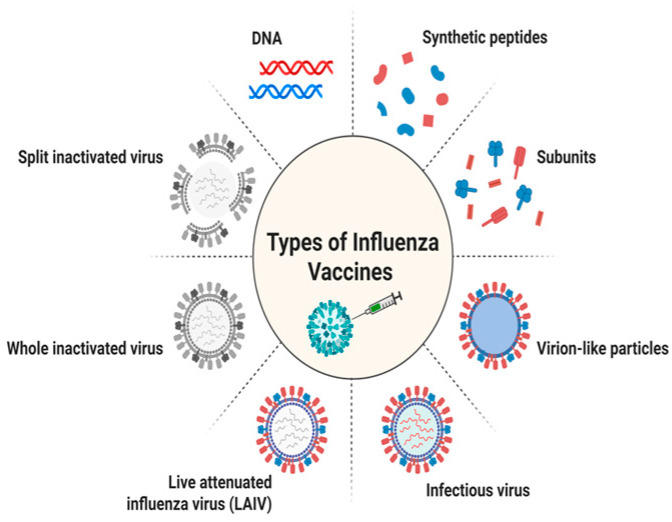
Various types of influenza vaccine. Image was created by BioRender.

**Figure 3:**
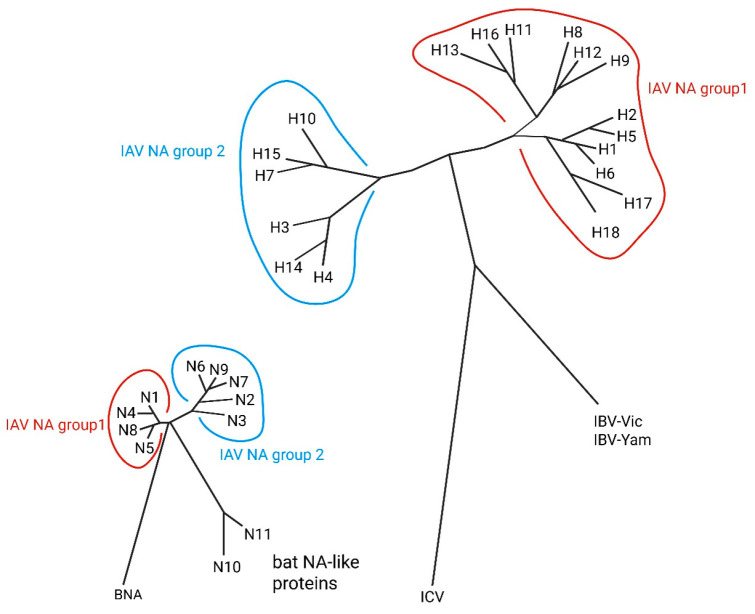
Phylogenetic tree of influenza antigens. Image was created by BioRender.

To date, influenza vaccination is still the most powerful public health measure and can not only help stop the spread of flu but also cut down any linked health issues and deaths. Presently, the three kinds of influenza vaccines that people commonly use are inactivated influenza vaccine (IIV), live attenuated influenza vaccine (LAIV), and recombinant influenza vaccine (RIV). Various types of influenza vaccine are represented in Figure 2 and a phylogenetic tree of key influenza antigens in Figure 3.

### 3.1. Inactivated Vaccines

Inactivated influenza vaccines (IIVs) have been manufactured and utilized since the 1940s and represent the most widely produced and used type of influenza vaccine. The main components of these vaccines are hemagglutinin and neuraminidase proteins; some specific formulations may also have nucleoprotein, either with or without an adjuvant that is added [36].

The viruses present in these vaccines are inactivated, meaning they cannot cause influenza; however, mild side effects might occur, such as localized reactions where the injection was administered. IIV is approved for individuals 6 months of age and older, including pregnant women and those with chronic health conditions. A single dose is recommended to be administered into the deltoid muscle or thigh. However, for children between 6 months and 8 years who did not receive the seasonal influenza vaccine in the previous season, it is advised to give two doses at least 4 weeks apart. Receiving the influenza vaccine during pregnancy offers protection for both the mother and her newborn against influenza [37]. Despite its long history of usage and dominance, inactivated influenza vaccines (IIVs) have several inherent drawbacks. Their poor stimulation of T-cell-mediated immunity, especially that of cytotoxic T lymphocytes (CD8+), and their failure to confer cross-protection against antigenically drifted or heterologous strains is a significant drawback [38]. Moreover, this egg-based production has a potential risk of becoming vulnerable to egg adaptive mutations and, hence, antigenic match and inefficient vaccines, especially in the hemagglutinin (HA) protein, which has been of great concern to the H3N2 strains. The production course in itself is long and normally takes a period of up to six months, and this slows the response rate of the platform to emerging or pandemic strains [39]. Moreover, according to pediatric dosing, two doses are also required in children who have not received any vaccination but are aged between 6 months and 8 years, which can adversely affect the compliance of vaccination in this age category [40].

### 3.2. Live Attenuated Vaccines

Live attenuated influenza vaccines (LAIVs) consist of attenuated viruses and do not cause the flu but can cause mild symptoms, like a runny nose, nasal congestion, fever, or sore throat. Side effects of the vaccine are generally mild and temporary, in comparison to those of an influenza infection. LAIV is recommended for use in persons aged 2–49 years who do not have contraindications or precautions but should not be given to pregnant women. LAIV is given as a nasal spray and is only one dose, but children 2–8 years old who have not received the seasonal flu vaccine in the past should get two doses, at least 4 weeks apart [37]. Live attenuated influenza vaccines (LAIVs) are beneficial in their mucosal immunization capacity, but they are limited by various constraints of their platforms. Such vaccines are thermolabile, and the cold chain requirements must be highly maintained; this becomes an issue for large-scale distribution, especially in low-resource settings [41]. They cannot be used in immunocompromised people and pregnant women, which limits the target population in which they can be used [39]. LAIVs have not been consistent across seasons and among all age groups, and in adults, they have been worse than in children. The risk remains mostly theoretical, but there is the concern of the possible reversion of the attenuated virus to a more virulent form, particularly in immunocompromised hosts, which warrants strict post-marketing surveillance.

### 3.3. Recombinant Vaccines

Recombinant influenza vaccines (RIVs) deliver the hemagglutinin (HA) protein of the influenza virus, the principal immunogenic protein for this disease, to the body. An RIV is manufactured in insect cells with a baculovirus expression vector system. It is licensed for use in adults ≥18 years of age and has shown superior efficacy against PCR-confirmed influenza illness compared with an egg-based standard-dose vaccine in this population [42]. Different vaccines under clinical trials are given in Table 1. The benefits of using recombinant influenza vaccines (RIVs) are that they do not rely on eggs, and they can be scaled up much quicker. The main issue with the usage of RIVs is that they only express the HA antigen and lack neuraminidase (NA) and internal viral proteins, which limits the scope of response and the strength thereof [43]. Such vaccines are also quite costly to manufacture due to the complexity of the insect-cell-based manufacturing process in which baculovirus vectors are used [44]. Recombinant vaccines are also prone to a shorter shelf life and vulnerability to unfavorable environmental stress compared to the conventional platforms. Additionally, when administered intramuscularly, they do not give rise to potent mucosal immunity, which is especially important when it comes to airway pathogens, such as influenza [36]. These drawbacks have spawned more universalized vaccines, such as the mRNA-based and the nanoparticle-based ones.

## 4. Influenza Vaccine Effectiveness

The evaluation of influenza vaccines depends on two fundamental criteria, which are efficacy and effectiveness. The measurement of vaccine efficacy demonstrates how vaccination decreases the chances of developing infections and related diseases, which occurs in randomized, placebo-controlled clinical trials. The concept of vaccine effectiveness determines how much vaccinated people experience reduced clinical outcomes, such as disease incidence and hospital admissions, in real-world scenarios analyzed through observational studies [52].

Randomized placebo-controlled clinical trials are established as the highest standard for determining vaccine effectiveness; however, their implementation involves high costs and may generate moral dilemmas. Influenza vaccine efficacy trials often encounter challenges with effectiveness due to fluctuations in infection rates and the mismatch between vaccine strains and seasonal virus strains. Observational studies are becoming an increasingly viable solution for examining vaccine effectiveness (VE) due to practical limitations [53]. The test-negative design (TND) has emerged as the leading vaccine evaluation method since the start of the 2004–2005 influenza season. The method uses influenza testing to separate participants into two groups: positive tests determine cases, and negative tests designate controls [52].

Current influenza vaccines achieve effectiveness levels that do not reach optimal standards since they typically protect between 40 and 60 percent of cases when the vaccine strains match the current circulating viruses. Different factors influence the effectiveness of a vaccine, such as the age of the recipient, along with the degree of alignment of the vaccine strain with circulating viruses and potential egg-based vaccine modifications, and the individual’s past receipt of influenza vaccinations [54]. Vaccines compared based on effectiveness in the past five years is represented in the given Table 2.

Annual re-administration is required for influenza vaccines due to high probabilities or risk of circulating virus mutation (antigenic drift), rapidly reducing the level of antibodies induced by influenza vaccine with time. Influenza vaccination has only short-term benefits because of the limited vaccination protection up to only the next epidemic, requiring repeated influenza vaccination annually, which is recommended in numerous countries [55].

**Table 2:** Vaccine compared based on effectiveness in the past 5 years.

Season	Vaccine Type	Target Strain(s)	Effectiveness (%)	Age Group	Study Type	References
2019–2020	Inactivated (IIV4)	H1N1pdm09, H3N2, B/Victoria, B/Yamagata	~45%	All adults	TND (CDC)	[56]
2020–2021	Recombinant (RIV4)	Same as above	~51%	18–49 yrs	Clinical trial	[57]
2021–2022	Live Attenuated (LAIV)	H3N2 mismatch season	~25%	2–17 yrs	TND (US/UK)	[58]
2022–2023	Cell-based IIV	H1N1, H3N2	~47%	Elderly	TND (CDC)	[59]
2023–2024	mRNA Influenza (Trial)	H3N2, B/Vic	~63%	18–64 yrs	Phase II/III trial	[60]

IIV4: Inactivated influenza vaccine, quadrivalent, RIV4: recombinant influenza vaccine, quadrivalent, LAIV: live attenuated influenza vaccine, TND: test-negative design (a type of observational study commonly used to assess vaccine effectiveness), CDC: Centers for Disease Control and Prevention, mRNA: messenger ribonucleic acid, H1N1pdm09: pandemic H1N1 influenza A virus strain from 2009, H3N2: influenza A virus subtype H3N2, B/Victoria: influenza B virus, Victoria lineage, B/Yamagata: influenza B virus, Yamagata lineage, Phase II/III trial: combined phase II and phase III clinical trial (evaluates safety, dosing, and efficacy in larger populations).

## 5. Challenges in Vaccine Development

### 5.1. Antigen Drift and Shift

**Figure 4:**
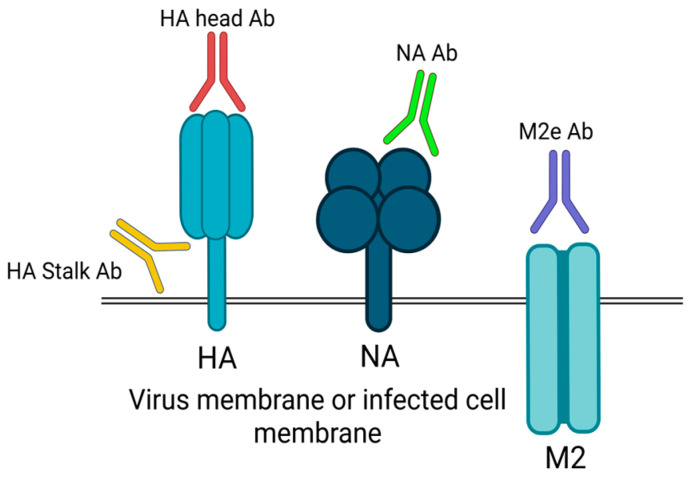
The antigenic drift of influenza viruses, primarily due to the high frequency of mutations and genetic recombination of genes encoding proteins associated with the surface of viruses, such as hemagglutinin (HA) and neuraminidase (NA), forming new viral strains that may escape pre-existing immunity [65]. This instability makes it hard to develop vaccines because they need to be altered often to match the latest strains. The image shows that antibodies may bind to varying parts of viral proteins, including the HA head, HA stalk, NA, and M2e. Due to the hypervariability of the head of HA, most neutralizing antibodies are strain-specific, although targeting more uniform areas, like the stalk of the HA, the NA, or M2e, can elicit a broader and potentially universal protection. There has therefore been a shift in vaccine development towards approaches that target those conserved sites to bypass the antigenic diversity to generate more protective and long-term immunity [65]. Image was created by BioRender.

Influenza viruses are constantly changing to hemagglutinin (HA) and neuraminidase (NA) proteins as a result of antigenic drifts (Figure 4); thus, seasonal influenza vaccines have to be routinely updated to include new viral antigens. The virus must have these two proteins to infect the cells well: HA allows cells to adsorb viruses and enter the cell, and NA ensures the cell releases other viruses and decreases the aggregation of viral particles [61]. The available chains of influenza vaccines largely induce the production of antibodies against hemagglutinin, with some also containing neuraminidase or other viral proteins [62].

Regardless of the antigenic drift, influenza A and B viruses differ in evolutionary rate as they go through their lifecycle. The evolutionary rate of the H3N2 viruses is more direct than with influenza B and H1N1 viruses and leads to rapid changes. Scientists suppose that influenza B viruses have a slower genetic change rate of two to three times when compared to influenza A [63]. During the shift of influenza B viruses among hosts, they portray a polymerase enzyme that exhibits low mutation rates in comparison with other strains and a broader genetic bottleneck. The precise reasons that they evolved more slowly are as yet unknown until today. The major factor influencing the H3N2 virus consists of a strong antigenic drift that often causes the premature development of harsh influenza seasons [64].

### 5.2. Vaccine Strain Selection

The initial global influenza surveillance system came into existence through the World Health Organization in 1952 [66]. The World Health Organization uses multiple criteria to determine vaccine strain selection, which include worldwide surveillance results and antigenic information, and suitable vaccine virus strains at a ratio of 50 percent [67]. Influenza vaccine selection is an important process that involves selection of a specific strain of influenza virus needed to be included every year in the seasonal influenza vaccine. Seasonal vaccines contain 3–4 viral strains (one H3N2 influenza A, one H1N1 influenza A, and one or two influenza B viruses), and WHO, twice a year, recommends the strain to be included in the seasonal vaccine. Every year, thousands of viral isolates and antigenic properties are evaluated by the Global Influenza Surveillance Network [67]. Influenza isolates traditionally undergo hemagglutination inhibition (HAI) testing to measure their antigenic properties, yet this method has given way to virus neutralization testing, which is more frequently used today because current H3N2 strains do not effectively agglutinate red blood cells [67]. Due to the mutation of viral hemagglutinin (HA), primarily in the receptor-binding site, which binds to sialic acid receptors on red blood cells and might not effectively detect the antigenicity of the virus, the HAI assay is less sensitive [68]. The massive viral sequence data are investigated and take into account how specific variants are geographically distributed during vaccine strain selection. Human immune system complexity leads to the production of diverse antibodies by humans and ferrets. Antisera are required for antigenic characterization of viral isolates. Examining human vaccination sera has become a standard laboratory practice for enhancing the characterization of influenza virus antigenic properties alongside ferret antisera [63]. For many years, since the 1950s, vaccine strain selection has been performed by serological assay, although with several disadvantages, like being labor-intensive, not publicly available, difficulty in scaling up, and being hard to interpret or unreliable across labs [69].

Identification of the common antibody targets in individuals of different ages with diverse immune histories is important in scientific studies. Individuals have immune strategies that focus on epitopes (in the HA) of the initial childhood strains of the virus [70]. Failure in vaccines happened in the 2013–2014 influenza season due to the existence of vaccines whose features were age-specific among individuals vaccinated between 1977 and 1985. These pieces of information can be identified by researchers by examining the samples of human serum. In assessing new strains of the influenza virus, researchers ought to sample sera of individuals in various age groups [63].

Despite periodic modifications, vaccine manufacturers struggle to precisely align their products with the existing influenza virus types. The vaccine industry needs to forecast the prevalent strains of each flu season several months in advance because the current manufacturing timeline extends up to six months for vaccine development [71].

A universal vaccine would target the conserved regions, provide long-lasting protection against the virus, and would probably require no annual reformulation. A successful universal flu vaccine would provide extended immunity against all strains, irrespective of antigenic shift or drift or virus subtypes [72].

### 5.3. Production Limitation

Most influenza vaccines (over 80%) undergo egg-based production methods, which require six months before completion and expose the process to supply chain breakdowns while creating egg-adaptive mutations that reduce vaccine effectiveness. Modern production methods, which include cell culture and recombinant technologies, provide quicker output times and avoid egg-related problems, yet they encounter limited production capacity and incur higher expenses. The global distribution of vaccine manufacturing facilities remains unequal because several major regions, including Africa, do not possess local production capabilities, which causes distribution problems during seasonal epidemics and pandemics. Global vaccine availability will improve through the essential development of new manufacturing facilities, particularly in low- and middle-income countries [73].

The development of advanced vaccines faces difficulties related to increased scale operations, compliance requirements, and community resistance. The potential of mRNA vaccines and nanoparticle-based platforms requires extended verification, in addition to product refinement, for their successful implementation [74].

## 6. Impact of COVID-19 on Influenza Vaccination

Higher levels of vaccination were noted in 2020 when compared to previous influenza seasons, especially among the older population, healthcare workers (HCWs), and persons with chronic medical conditions. The increased vaccination rates were the result of better observance of precaution measures that were undertaken in the current pandemic and the better evaluation of the risk of influenza. Research shows that more COVID-19 consequences were associated with much higher influenza immunization rates among healthcare workers since they wanted to secure themselves and patients [75,76].

General population rates of influenza vaccination have been variable, sometimes falling after the pandemic despite the initial impression of rising. Vaccine depletion, distribution problems, and the feeling that the risk of influenza had decreased also lowered influenza vaccination in several countries after the pandemic compared to before the pandemic. In the post-pandemic era, there was a decreasing rate of influenza vaccination in the general population of Saudi Arabia compared to that before the pandemic, i.e., 43.3% down to 29.1% [76].

The healthcare system became overwhelmed as a result of the epidemic, and influenza vaccine efforts received even greater challenges. The provision of influenza and COVID-19 vaccines has to be thoroughly planned to avoid excessively overloading healthcare resources and to comply with demands of the recommended vaccine intervals. To sustain influenza vaccination coverage in the pandemic, important emphasis shifted to a smaller number of areas: the availability of vaccines in a convenient environment and, more importantly, their safety and easy access; and the implementation of tailor-made communication strategies [77]. Masks and social separation were two of the COVID-19 control techniques that led to record-breaking decreases in influenza transmission in 2020 and 2021. Vaccination programs are necessary to restore herd immunity levels because the decreased viral circulation in the population led to a drop in the development of natural immunity in children and other groups [78].

## 7. Role of Nanotechnology in Vaccine Development

Human vaccines are developed with low pathogenicity and high antigenicity by using the altered forms of bacteria and viruses. Live attenuated bacterial vaccines have mutation problems, and killed vaccines need adjuvants, like polymers, mineral salts, and emulsions, to boost vaccine action by upregulating cytokines, sustained antigen release, or activating antigen-presenting cells [79]. Different research groups have focused on the development of new antigenic agents and methods of administration, mainly to tackle anaphylactic reactions and reactivation of microorganisms [80]. In vaccinology, the application of nanotechnology has increased in the past few decades [81]. The medical field has advanced due to the application of nanotechnology and the design of nanostructures, which improve health by repairing cells after administration in the human body. Early vaccine problems are being addressed by nanotechnology [82]. Nanovaccines assist the immune system in fighting infections by tiny particles called nanoparticles. Nanovaccines are recognized by dendritic cells and macrophages, start generating signals known as cytokines, and aid in initiating the defense mechanism by attracting additional immune cells [83]. As the subsequent first response, the body triggers the powerful and specific adaptive immune system. Immune cells engulf nanovaccines and deliver the vaccine components/antigens to specialized T cells. The T cells destroy infected cells with the aid of CD8+ T cells; B cells produce antibodies with the aid of CD4+ T cells, and the developed antibodies also protect the body from future infections.

**Figure 5:**
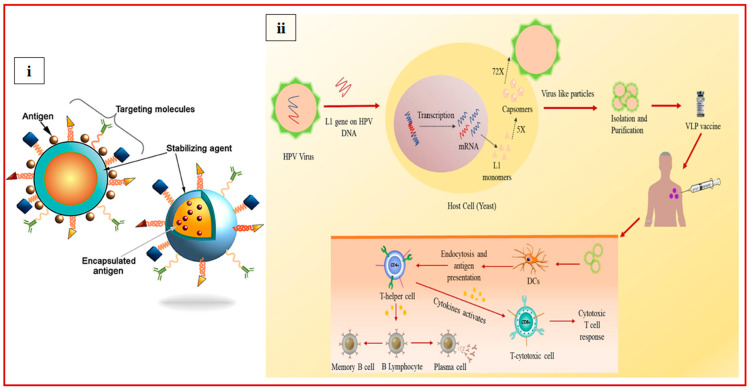
(**i**) Schematic representation of the nanocarriers [85] (copyright © 2018 Pati, Shevtsov and Sonawane, https://creativecommons.org/licenses/by/4.0/). (**ii**) Schematic diagram illustrating the VLP-based vaccine design for human papillomavirus (HPV) and the corresponding immune response [86], adapted with permission from copyright © 2023 Elsevier Inc.

Nanoparticles less than 1000 nm are used in vaccines for prophylactic measures. Nanoparticles promote antigen processing or trigger immunity, and due to their small size, nanoparticles move through the cellular machinery by endocytosis and transport the active moiety alongside them [81]. Efficient influenza vaccines elicit specific antibody responses; for example, immunoglobulin G (IgG) affects the functioning of HA by inhibiting fusion or obstructing the binding of the host receptor. Various types of nanoparticles are being used in the development of the influenza subunit vaccine, such as virus-like particles (VLPs), bacterial spores, polysaccharides, bacteriophages, immune-stimulating complexes (ISCOMs), liposomes, virosomes, and inorganic NPs. Nanovaccines use nano-sized carriers (10–1000 nm) to encapsulate and deliver antigens, adjuvants, or genes, as shown in Figure 5i [84].

## 8. Various Types of Nanoparticles

### 8.1. Natural Nanoparticles

#### 8.1.1. Virus-like Particles (VLPs)

Virus-like particles (VLPs) are non-replicating and self-assembling in nature without any infectious genetic material. VLPs can be used as immunopotentiators or particulate carriers in the process of vaccine development because of their immunogenic characteristics, like size similarity with the pathogen, repetitive geometry of the surface, and the ability to provide potent antibody responses and B-cell activation. They can be produced from yeast, bacteria, animal cells, and insects as host cells [87,88]. Cervarix^®^, Gardasil^®^, and Gardasil9^®^ are licensed vaccines for human papillomavirus (HPV), and VLP-based HPV vaccines are shown in Figure 5ii. A VLP-based approach can be used for the development of a universal influenza vaccine. Efficient VLP vaccine development requires selecting an appropriate VLP construct and integrating the antigen without destabilizing VLPs [89]. H3N2-VLPs expressing NA, M1, and HA proteins induce stronger protective antibody responses as compared to whole inactivated vaccines in mice [90]. At phase 2 clinical trials, Nanoflu, a quadrivalent VLP with adjuvant Matrix-M, was tested in ≥65-year-old adults and reported to elicit potential HAI responses [91]. Recombinant technology is used to modify the VLP by adding more adjuvants, like bacterial protein, to enhance its immunogenic nature. After adding adjuvant Salmonella flagellin protein to VLP with M1 proteins and HA, the levels of IgG2a, IgG2b, and cytokine responses were observed to be higher compared to the control VLP deficient in Salmonella flagellin. The chimeric VLPs offered full protection against the PR8 virus and achieved a 67% survival rate against a lethal dose of the heterosubtypic H3N2 strain [92]. Phage VLPs are non-immunogenic, and no previous immunity against them exists in humans, which makes them safer compared to others. Bacteriophage VLPs utilize the capsid proteins of phage to present peptides or proteins, with the cargo size depending on the type of phage [93]. In a recent study, through genetic engineering, influenza-conserved nucleoprotein (NP) was incorporated into bacteriophage P22, and mice (vaccinated) produced anti-NP antibodies, as well as CD8+ T-cell responses (NP-specific). Intranasally administered vaccines protected mice against H1N1 and H3N2 viruses [94].

#### 8.1.2. Bacterial Spore

Bacterial species produce certain dormant cells, which are known as spores [95]. Bacteria can withstand harsh environments by forming 800–1200 nm spores of spherical/ellipsoidal shape [96]. A spore has the ability to act as a vaccine carrier, as it can self-assemble into its functional structure and protect the antigens on its surface, eliciting an immune response. The antigen can be protected from the harsh acids of the stomach after oral administration before reaching the immune cells of the gut [97]. By using recombinant technology, bacterial spores are conjugated with vaccine antigens of appropriate size and complexity for presentation of an antigen [98]. The spore coat protein of B. subtilis PY79 was fused with three replicates of matrix protein M2e to use for an oral influenza vaccine design. Repeated immunization elicited strong cellular immune responses and M2e-specific IgG (titer of 1:12,800 at week 17 post-first vaccination). In addition to a 100% survival rate, lung specimens from inoculated mice that were subsequently challenged with the A/PR/8/34 (H1N1) influenza virus showed noticeably lower levels of the viral titers than those from the control group [99]. B. subtilis spores, both live and heat-inactivated, can be directly used for vaccine development because of their ability to bind influenza virions. In a study, mice immunized intranasally with killed spores adsorbed to H5N1 virions were completely protected, even from a lethal dose of the virus [100].

#### 8.1.3. Polysaccharide

Polysaccharides are safe, biocompatible, and biodegradable, along with having an immunomodulatory action. Polysaccharides are polymers of natural origin with carbohydrate monomers linked glycosidically [101]. Chitosan and its derivatives are polysaccharides commonly used as vaccine adjuvants, ascribed to their capability to activate the immune system and promote antigen-specific immune responses [102]. Influenza DNA vaccines have been developed using chitosan NPs and have been found to have a high encapsulation rate and stability. It was stated that DNA-loaded chitosan NP vaccines prolonged the release of the DNA plasmid with stronger immune responses than with the DNA vaccine alone [103].

#### 8.1.4. Outer Membrane Vesicles (OMVs)

Outer membrane vesicles (OMVs) are nano-sized spherical structures that Gram-negative bacteria spontaneously discharge from their outer membranes. These vesicles are typically 20 to 250 nanometers in diameter and consist of a lipid bilayer loaded with proteins, lipopolysaccharides (LPS), phospholipids, and periplasmic components. OMVs are released by bacterial cells in reaction to environmental stressors, such as antibiotic exposure, temperature changes, and host immunological responses [104]. OMVs act as carriers for bacterial virulence and can be transferred directly to host cells [105].

Furthermore, OMVs can be modified to carry specific antigens, detoxified to remove harmful LPS, and designed for injection, oral, or intranasal delivery. Their flexibility allows for the development of vaccines that guard against different infectious pathogens, including viruses and bacteria. Several approved vaccines have effectively used OMV-based platforms, particularly those that prevent meningococcal disease [106]. Because of their intrinsic adjuvanticity, safety, and capacity to replicate natural infection pathways without the dangers of live pathogens, OMVs are being investigated for next-generation vaccines as research progresses. Shehata et al. developed a bacterial OMV-based vaccine presenting the stable antigenic chimeric protein of the H1-type HA of the influenza A virus (H1N1) strain (H1N1pdm09) from the 2009 pandemic and the receptor-binding domain (RBD) of the Middle East Respiratory Syndrome Coronavirus—MERS-CoV (OMVs-H1/RBD) [107].

#### 8.1.5. Extracellular Vesicle Vaccines (EVs)

Nearly every type of cell, including immune, epithelial, and tumor cells, secretes extracellular vesicles (EVs), which are small particles surrounded by a membrane. These vesicles are usually classified into three groups based on their size and origin. The potential of EV-based vaccines to protect against various diseases, including bacteria, parasites, and viruses like influenza [108], coronaviruses, and HIV, is currently being explored [109,110]. Their membrane structure allows them to merge easily with target cells, which improves antigen delivery. Additionally, their natural makeup helps them avoid detection by the immune system. Despite their great promise, EV-based vaccines face several challenges in the clinical translation process. These challenges include ensuring uniform cargo loading and immune response, as well as the need for standardized methods for isolation, purification, and large-scale production. However, ongoing advancements in nanomedicine, biotechnology, and bioengineering are gradually addressing these issues. With additional study and advancement, EV-based vaccines have the potential to emerge as major contributors to next-generation vaccination methods, providing a highly adaptable, safe, and efficient platform for therapeutic and preventative immunization. In clinical studies, the proficiency of exosome-based vaccines to trigger innate immunity was demonstrated. But further investigation is essential to produce innovative methods for EV loading with particular drugs or antigens and to alter EVs to carry cargo more efficiently [111].

### 8.2. Synthetic Nanoparticles

#### 8.2.1. Inorganic NPs

**Figure 6:**
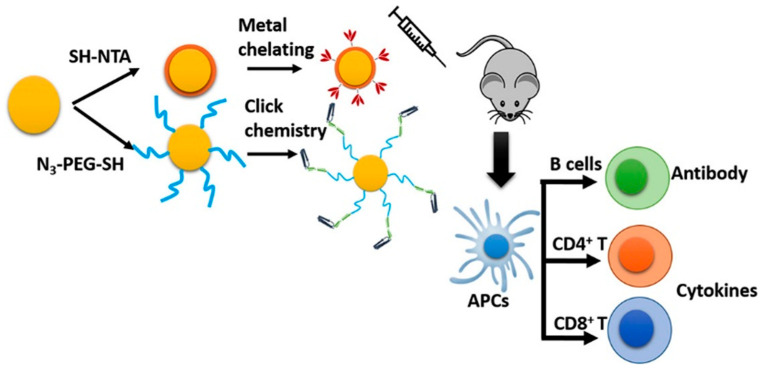
The immunogenicity of subunit vaccines can be increased by developing a nanoformulation [114] adapted with permission from copyright © 2018 Elsevier Inc.

Nanoparticles are typically assembled with an inorganic core and an organic shell layer, and recently, inorganic nanoparticles have attracted interest from various research groups for use in vaccines [112]. AuNPs have low toxicity, and their size, composition, shape, and surface functionalization make them ideal for vaccine application. Chen et al. studied various spherical AuNPs, ranging from 2 to 50 nm in diameter, conjugated with synthetic foot-and-mouth disease virus peptide [113]. Wang reported conjugated recombinant influenza A/Aichi/2/68 (H3N2) hemagglutinin (HA) onto functionalized AuNP, shown in Figure 6 [114]. Tao et al. demonstrated the ability of functionalized AuNPs with the extracellular domain of M2 peptide (M2e) to induce immunity against influenza A [115]. Wang C et al. reported that conjugating recombinant influenza hemagglutinin trimers and flagellin to gold nanoparticles enhanced cellular immunity (mucosal) [114]. Vaccine efficiency can be increased by using a promising approach of functionalization of AuNPs with carbohydrate conjugates used to enhance immunogenicity. Iron oxide nanoparticles are composed of Fe_3_O_4_ and γ-Fe_2_O_3_ magnetite, usually by thermal decomposition or oxidative coprecipitation. Several IONP-based platforms have been developed as vaccine adjuvants. Neto et al. synthesized Mycobacterium tuberculosis fusion protein-modified citrate-coated manganese ferrite (MnFe_2_O_4_) nanoparticles using coprecipitation [116]. Current initiatives have focused on the utility of iron oxide nanoparticles (IONPs) as an adjuvant for the development of vaccines. Inflammatory processes are initiated or activated in the presence of a critically important element, i.e., iron. For instance, Roja et al. synthesized superparamagnetic iron oxide nanoparticles (SPIONPs) modified with dimercaptosuccinic acid, aminodextran, or aminopropyl silane and evaluated them in M2 macrophage murine models [117]. Mesoporous silica nanoparticles (MSNPs) represent a highly promising platform for vaccine delivery because of their chemical stability, biocompatibility, and biodegradability [118]. Various research groups have utilized aminosilane-functionalized silica material (SBA-15) for the delivery of an antigen vaccine aiming to improve mechanistic, as well as immunogenic properties. Carvalho et al. studied SBA-15 for an adjuvant effect and an antibody response, with bovine serum albumin and aluminum hydroxide subcutaneously delivered in genetically modified mice [119].

#### 8.2.2. Liposomes

Liposomes are lipid bilayers containing an aqueous core for loading hydrophilic pathogenic antigens, or if they are hydrophobic, then in the bilayer (single- or multi-layered) [120]. The antigens can be present on the membrane surface in liposomes. The surface area of liposomes allows an antigen’s multivalent presentation. Adjuvants and multiple agents can be co-delivered by liposomes. The size, charge, number of antigens, and other parameters of liposomes can be adjusted to impact the trafficking and activation of the immune responses. Liposomes have a degradation problem in conditions like the immune system and varying pH and in the presence of enzymes [121].

#### 8.2.3. Polymer NPs

**Figure 7:**
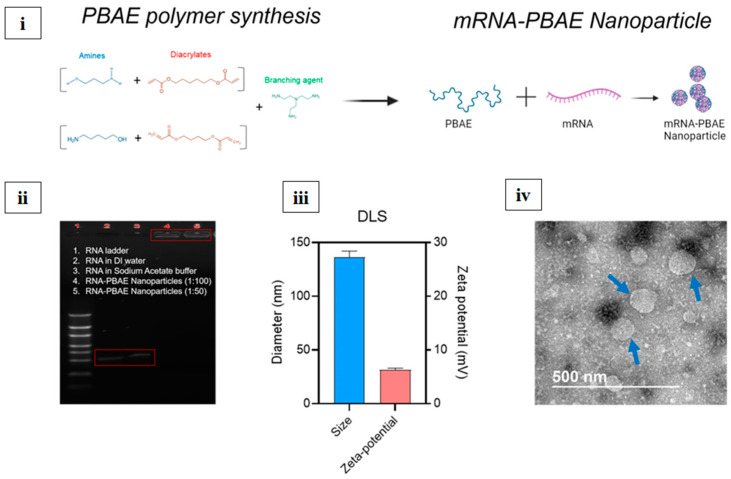
(**i**) Synthesis of PBAE polymer and nanoparticle fabrication. (**ii**) Image of gel electrophoresis assay. (**iii**) Size and zeta potential. (**iv**) TEM image of nanoparticles; blue arrows, scale bar = 500 nm [126]. Adapted with permission, copyright © 2023, The Author(s), http://creativecommons.org/licenses/by/4.0/.

Polymeric nanoparticles are composed of biocompatible polymers, mainly polyglycolic acid (PGA), poly (lactic-co-glycolic acid) (PLGA), and polylactic acid (PLA). These polymers could be altered in size, composition, and surface properties, resulting in controlled drug release and protection [122]. Thomas et al. synthesized nanoparticles by varying the ratio of PLA and PLGA to deliver the hepatitis B surface antigen for the treatment of hepatitis B virus (HBV) through the pulmonary route [123]. Polymers are flexible and tunable, and other polymers, such as chitosan, can be incorporated on the outer surface for efficient mucosal vaccine delivery [124]. Polymeric nanoparticles can act as a depot to gradually release the encapsulated antigen [125]. Kim et al. developed an mRNA vaccine nanoparticle using an mRNA and PBAE polymer, as shown in Figure 7i. The gel electrophoresis assay is shown in Figure 7ii, and the size and zeta potential of the nanoparticles are shown in Figure 7iii. TEM images of the nanoparticles are shown in Figure 7iv [126]. Currently, there is no FDA-approved polymeric nanocarrier for influenza, although it is in preclinical and clinical stages. Table 3 lists various types of nanoparticles.

**Table 3:** Types of nanoparticles.

Type of Nanoparticle	Antigen Used	Adjuvant	Route of Administration	Targeted Influenza Subtype	Trial Phase/Outcome	References
**VLP (Virus-Like Particle)**	Hemagglutinin (HA) from H1N1	Matrix-M™	Intramuscular (IM)	H1N1	Phase III (NanoFlu, Novavax)—Safe, strong immunogenicity	[45]
**Lipid Nanoparticle (LNP)**	mRNA-encoding HA (H1, H3, B-Yamagata, B-Victoria)	None (self-adjuvanting)	Intramuscular (IM)	Multivalent	Phase I/II (Moderna mRNA-1010)—Under evaluation	[49]
**Polymeric Nanoparticle (PLGA)**	Recombinant HA (rHA) from H5N1	MPLA	Intranasal (IN)	H5N1	Preclinical—Strong mucosal and systemic response	[81]
**Gold Nanoparticles (AuNPs)**	Full-length HA from H3N2	CpG ODN	Intranasal (IN)	H3N2	Preclinical—Enhanced Th1-biased immune response	[127]
**Chitosan Nanoparticle**	Conserved M2e peptide	TLR agonist	Intranasal (IN)	Universal target	Preclinical—Cross-protective, improved mucosal IgA	[128]
**Liposome-Based**	HA and NP proteins	Monophosphoryl lipid A	Subcutaneous (SC)	H5N1	Preclinical—High T-cell responses	[129]
**Ferritin Nanoparticle**	HA from H1, H3, B strains	None	Intramuscular (IM)	Multivalent	Phase I (NIH)—Ongoing; induces broadly neutralizing antibodies	[130]
**Micelle (Self-assembling)**	M2e peptide	TLR7 agonist	Intranasal (IN)	Broad/universal	Preclinical—Strong mucosal immunity, IgA, and IFN-γ	[131]
**Polyanhydride Nanoparticle**	Inactivated whole virus	None	Subcutaneous (SC)	H1N1	Preclinical—Prolonged antigen release	[132]

## 9. Challenges

### 9.1. Antigen Design Approaches (Universal Influenza Vaccine)

**Figure 8:**
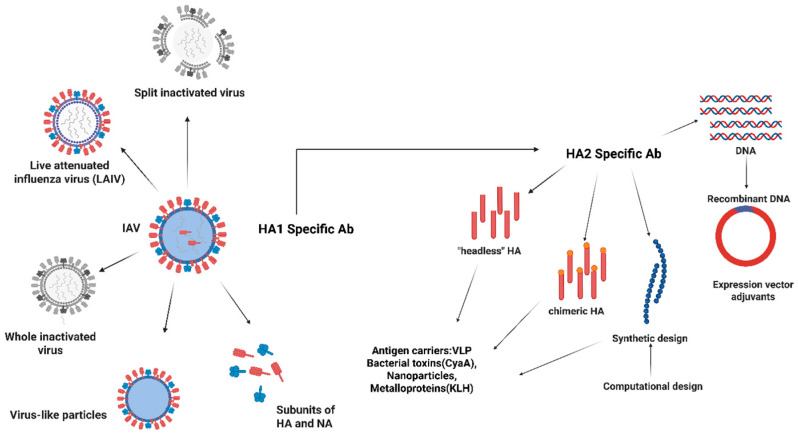
Diagrammatic representation of antigen design approaches. The figure shows the evolution of influenza vaccine strategies, from traditional techniques, such as whole inactivated virus vaccines, live attenuated virus vaccines, split inactivated virus vaccine, and HA and NA subunits of the virus, to the development of broader and universal vaccines that cover conserved sequences in the HA protein. Although existing vaccines primarily induce antibodies to the variable HA1 domain, new strategies target the conserved HA2 stalk domain [65]. The figure outlines the mechanisms by which novel approaches, such as the use of headless and chimeric HA constructs, computational and synthetic design, recombinant DNA, and various antigen carriers, are intended to enhance HA2 immunogenicity and induce broadly reactive antibodies. Such initiatives are highly crucial in advancing vaccines to deliver cross-protecting immunity among various strains of the influenza A virus, to overcome the problem of antigenic variation [138]. Image was created by BioRender.

One crucial future path is the development of a universal influenza vaccine, which would offer widespread and durable defense against a variety of influenza strains, including those with the potential to spread like a pandemic. Antigen design approaches (shown in Figure 8) focusing on enhancing cross-reactive immunity include (a) HA-based, (i) targeting conserved regions. Current influenza vaccines primarily target the highly variable hemagglutinin (HA) head domain to produce strain-specific immunity and provide a very narrow breadth for protective antibodies targeting the head domain. To provide both homosubtypic and heterosubtypic protection against a variety of influenza subtypes, universal vaccines seek to induce immunity against conserved viral components, including the HA stem and residues of receptor pockets. These domains are conserved even for subtypes, and therefore, neutralizing antibodies against them would be cross-protective. (ii) Chimeric HA (cHA)—In this approach, the same stalk domain and different head domain were used for cHAs, inducing anti-stalk Abs through successive vaccinations. (iii) Mosaic HA (mHAs)—This approach targets the conserved region to immunize against a wider range of strains and elicit an immune response against both head and stalk domains. mHAs are engineered HAs that have replaced the variable and immunodominant site from the head with other corresponding sites from influenza virus subtypes, mainly avian. mHAs can be used for sequential vaccination, with a conserved stalk and the framework of head domains, but immunodominant sites of head domains would be changed at every administration. (iv) COBRA (Computationally Optimized Broadly Reactive Antigens) HA—The COBRA strategy utilizes a computational method to make consensus sequences for entire strains from a specific HA subtype. (b) Matrix proteins (M1, M2)—Matrix protein M2 is a transmembrane protein with three prominent parts (the extracellular N-terminal (M2e), the transmembrane, and the intracellular C-terminal domain). M2 protein is essential for the uncoating process that occurs in endosomes. M2e is highly conserved but has low immunogenicity [133,134]. (c) Internal proteins, including matrix protein 1 (M1) and nucleoprotein (NP), are highly conserved influenza proteins. They are present inside the infected cells and are presented by the Major Histocompatibility Complex (MHC). Current influenza vaccines could be improved by this promising approach of cross-reactive T-cell response. (d) Multiple protein and (e) vaccine adjuvants are approaches to enhance vaccine effectiveness. Numerous universal vaccine candidates are undergoing clinical trials using a variety of platforms, such as nucleic-acid-based vaccines (DNA, mRNA), live attenuated viruses, recombinant proteins, synthetic peptides, virus-like particles (VLPs), and viral vectors. For example, the ability of ferritin-based nanoparticles presenting an HA stem, chimeric HA stem vaccines, and viral vectors generating NP and M1 proteins to generate broad and durable immunity is being evaluated [135,136]. These tactics also focus on increasing humoral and cellular immune responses to enhance cross-protection and pandemic preparedness [137].

### 9.2. Innovative Delivery Methods

Researchers are looking into new delivery methods to increase the accessibility, safety, and effectiveness of vaccines. Traditional egg-based production has two drawbacks: lengthy manufacturing durations and possible antigenic alterations during virus propagation. However, emerging technologies, such as mRNA vaccines, recombinant protein expression, and viral vector platforms, enable more rapid and flexible vaccine manufacturing. Plans are underway to create new immunization delivery platforms, like mRNA vaccines. The current COVID-19 and influenza vaccines offer rapid design and production capabilities, strong immune responses, and good safety profiles. The influenza and SARS-CoV-2 antigens are now included in a number of formulations to speed up immunization campaigns [139]. Vaccines employing virus-like particles (VLPs) and nanoparticle technologies improve antigen presentation and immune system activation, potentially leading to more thorough and long-lasting protection [137]. Advanced molecular engineering techniques can be used to develop new antigens and vaccine designs that more effectively elicit protective immune responses. Alternative routes, like intranasal sprays, oral vaccines, and microneedle patches, are being researched to increase immunization rates, lower costs, and enhance patient convenience and adherence [140].

## 10. Conclusions

To reduce the effects of both seasonal and pandemic flu, influenza vaccination is still a crucial part of public health initiatives. The current vaccines provide useful protection, but their production issues and strain-specific limits underscore the need for ongoing innovation. The goal of future research is to develop universal influenza vaccines that use cutting-edge vaccination technologies and target conserved viral components to produce widespread, long-lasting immunity. Modern delivery techniques, like mRNA platforms and enhanced antigen presentation systems, are anticipated to improve vaccine efficacy, speed up manufacturing, and expand accessibility at the same time. Nanotechnology has shown a new possibility for flu prevention and anti-influenza research, but the influenza virus rapidly and frequently modifies its genome, which poses a challenging task, and the mutation problem cannot be solved by the application of nanotechnology. Nanoparticles are excellent carriers to deliver nucleic acid, proteins/peptide, and vaccines, but they need to be check for their safety concerns for humans, as well as for the environment. Each nanocarrier system needs to be evaluated for its cytotoxic effects, drug interactions, and several other properties to utilize the complete potential of nanocarriers. By simplifying vaccination strategies, boosting immune responses, and strengthening preparedness for upcoming pandemics, these developments could collectively significantly improve influenza prevention and, eventually, lower the global burden of influenza. In the past few years, technology has achieved a magnificent achievement as a vaccine delivery platform due to its ability to prevent premature release of antigens and prolong the antigen presentation.

## Figures and Tables

**Table 1:** Different vaccines under clinical trials.

Product Name	Developer Company	Disease	Nanocarrier System	Viral Antigen Cargo	Clinical Trial (Registration Number)	Marketing Authorization	Ref.
NanoFlu	Novavax	Influenza	Virus-like particle (VLP)	rHA (H1N1, H3N2, B-Yamagata, B-Victoria)	Phase III (NCT04120194)	No	[45]
IVX-411	Icosavax	SARS-CoV-2	VLP (I53-50 scaffold)	SARS-CoV-2 RBD trimer	Phase I/II (NCT05027932)	No	[46]
ABNCoV2	AdaptVac	SARS-CoV-2	Capsid VLP (cVLP)	SARS-CoV-2 RBD	Phase II (NCT04839146)	No	[47]
BNT162b2	Pfizer/BioNTech	SARS-CoV-2	Lipid Nanoparticle (LNP)	mRNA-encoding SARS-CoV-2 spike protein	Phase III (NCT04368728)	Yes (Emergency Use)	[48]
mRNA-1010	Moderna	Influenza	Lipid Nanoparticle (LNP)	mRNA-encoding HA (H1, H3, B-Yam, B-Vic)	Phase I/II (NCT04956575)	No	[49]
Ferritin-HA	NIH/VRC	Influenza	Ferritin Nanoparticle	Hemagglutinin (H1, H3, B)	Phase I (NCT03814720)	No	[50]
Vaxfectin^®^ DNA vaccine	Vical Inc.	Influenza	Cationic lipid (Vaxfectin^®^)	DNA plasmid encoding HA	Phase I (NCT00709877)	No	[51]

## Data Availability

Not applicable.
